# Mechanisms Involved in the Anti-Inflammatory Action of a Polysulfated Fraction from *Gracilaria cornea* in Rats

**DOI:** 10.1371/journal.pone.0119319

**Published:** 2015-03-25

**Authors:** Chistiane Oliveira Coura, Ricardo Basto Souza, José Ariévilo Gurgel Rodrigues, Edfranck de Sousa Oliveira Vanderlei, Ianna Wivianne Fernandes de Araújo, Natássia Albuquerque Ribeiro, Annyta Fernandes Frota, Kátia Alves Ribeiro, Hellíada Vasconcelos Chaves, Karuza Maria Alves Pereira, Rodrigo Maranguape Silva da Cunha, Mirna Marques Bezerra, Norma Maria Barros Benevides

**Affiliations:** 1 Department of Biochemistry and Molecular Biology, Federal University of Ceará, Fortaleza, Ceará, Brazil; 2 Faculty of Medicine, Federal University of Ceará, Sobral, Ceará, Brazil; 3 Faculty of Dentistry, Federal University of Ceará, Sobral, Ceará, Brazil; 4 Biotechnology Nucleus of Sobral, Sobral, Ceará, Brazil; INSERM, FRANCE

## Abstract

The anti-inflammatory mechanisms of the sulfated polysaccharidic fraction obtained from red marine alga *Gracilaria cornea* (Gc-FI) were investigated using a paw edema model induced in rats by different inflammatory agents (carrageenan, dextran, serotonin, bradykinin, compound 48/80 or L-arginine). Gc-FI at the doses of 3, 9 or 27 mg/kg, subcutaneously - s.c., significantly inhibited rat paw edema induced by carrageenan and dextran, as confirmed by myeloperoxidase and Evans’ blue assessments, respectively. Gc-FI (9 mg/kg, s.c.) inhibited rat paw edema induced by histamine, compound 48/80 and L-arginine. Additionally, Gc-FI (9 mg/kg, s.c.) inhibited Cg-induced edema in animals with intact mast cells but did not inhibit that with degranulated mast cells by compound 48/80, revealing a protective role on mast cell membranes. Gc-FI down-regulated the IL-1β, TNF-α and COX-2 mRNA and protein levels compared with those of the carrageenan group, based on qRT-PCR and immunohistochemistry analyses. After inhibition with ZnPP IX, a specific heme oxygenase-1 (HO-1) inhibitor, the anti-inflammatory effect of Gc-FI was not observed in Cg-induced paw edema, suggesting that the anti-inflammatory effect of Gc-FI is, in part, dependent on the integrity of the HO-1 pathway. Gc-FI can target a combination of multiple points involved in inflammatory phenomena.

## Introduction

Inflammation is an organism’s protective reaction to several stimuli, such as microbial infection, chemical irritants and tissue injury. The early phase of acute inflammation involves the cellular influx associated with the release of mediators such as histamine and serotonin, which are primarily released from mast cells, followed by the production of bradykinin and prostaglandins. During an inflammatory response, several proinflammatory mediators are released, including interleukin-1 beta (IL-1β), tumor necrosis factor alpha (TNF-α) and cyclooxygenase-2 (COX-2). These mediators have important functions in the initiation and amplification of inflammatory processes [1]. One of the mechanisms involved in the resolution of inflammation is the expression of the enzyme hemoxigenase-1 (HO-1), which is responsible for catalyzing heme into carbon monoxide (CO), biliverdin/bilirubin and free iron. These products reduce inflammation and prevent the development of inflammatory diseases [2]. Although synthetic drugs are widely employed for controlling inflammatory disorders, adverse effects (e.g., gastric perforations, stomach ulcers and cardiovascular complications) are commonly observed [3], leading to increasing interest in research on the development of alternative tools that exhibit novel mechanisms of action from different origins [4, 5].

Seaweeds are rich sources of sulfated polysaccharides, which comprise an intrinsic group of highly complex and heterogeneous biopolymers that naturally occur in the extracellular matrix of these organisms [6]. These compounds are recognized as having several biological properties, including antioxidant [7], antithrombotic [8], gastroprotective [9], antinociceptive and anti-inflammatory [10] effects.

The sulfated polysaccharides that are present in red seaweeds are known as galactans. The enantiomeric variations, D- or L-, in the 4-linked α-galactose are named “carrageenan” or “agaran” [6].

*Gracilaria cornea* J. Agardh (Gracilariaceae, Rhodophyta) has been studied as a promising source of agar-type galactans. Melo et al. [11] previously identified the chemical structure of this polysaccharide by FT-IR and NMR techniques. 3,6-Anhydro-α-L-galactose has been identified as the main structural component; however, as minor components, 6-*O*-methyl-galactose, glucose, xylose and sulfated groups were also observed. Subsequently, Coura et al. [10] reported that its total sulfated polysaccharide (Gc-TSP) showed *in vivo* antinociceptive and anti-inflammatory effects without toxicological significance. This study analyzed the mechanisms involved in the anti-inflammation action of the polysaccharidic fraction from this algal species.

## Materials and Methods

### Animals

Wistar rats (180–240 g) from the Animal Care Unit of the Federal University of Ceará, Fortaleza, Brazil, were used throughout the experiments. This study was conducted in strict accordance with the guidelines set forth by the U.S. Department of Health and Human Services, and with the approval of the Ethics Committee of Animal Experiments of the Federal University of Ceará, Fortaleza, Brazil (Permit Number: 80/10).

### Isolation of sulfated polysaccharides

Specimens of *Gracilaria cornea* were collected along Flecheiras Beach, Brazil, at coordinates 03°13′06" S 39°16′47" W. A voucher specimen (no. 34739) was deposited in the Herbarium Prisco Bezerra of the Department of Biological Sciences, Federal University of Ceará, Brazil. The Instituto Brasileiro do Meio Ambiente e dos Recursos Naturais Renováveis (IBAMA) is federal organ responsible by protection of environment. Moreover, this study did not involve endangered or protected species. In according to the law MP 2.186–16/2001, Resolution n° 29 of Dispatch Component of Genetic Patrimony (CGEN), specific permissions no were required for use of seaweed *Gracilaria cornea* in this study. The algal samples were cleaned of epiphytes, washed with distilled water and stored at -20°C until use. Gc-TSP was obtained by protease digestion (60°C, 6 h) in 100 mM sodium acetate buffer (pH 5) containing EDTA and cysteine (both 5 mM) [12], with some modifications previously described by Coura et al. [10]. Gc-TSP (30 mg) was dissolved in 15 mL of 50 mM sodium acetate buffer (pH 5) and applied to a DEAE-cellulose column (1.5 × 21.5 cm) equilibrated with the same solution. The column was developed using a stepwise gradient of 0 to 2 M NaCl at 0.25-M intervals in the same solution. Fractions of 6 ml were collected and analyzed for sulfated polysaccharides using the metachromatic assay (A_525 nm_) containing dimethylmethylene blue with an Amersham Biosciences Utrospec 1100 spectrophotometer at 525 nm [13]. The metachromatic fractions were then dialyzed and freeze dried. The TSP and fractions obtained were analyzed by 0.5% agarose gel electrophoresis [14]. The biological protocols were performed with the fraction that showed the highest yield (named Gc-FI).

### Infrared (IR) spectroscopy

The Gc-TSP and obtained fractions were also characterized by IR spectroscopy. Fourier-transformed IR spectra were recorded using a Shimadzu IR spectrophotometer (model 8300) between 500 and 4000 cm^-1^. The samples were analyzed as a KBr pellet.

### Anti-inflammatory effect

#### Effect of Gc-FI on paw edema induced by dextran

Dextran (500 μg/paw, 100 μL), a classical osmotic agent [15], was injected s.c. into the right paws of rats. Animals (n = 6 per group) were treated with Gc-FI at the doses of 3, 9 or 27 mg/kg (0.1 mL/100 g body weight, s.c.) 1 h before the stimulus. Control animals received the same volume of sterile saline (0.9%, w/v, NaCl, s.c). The paw volume (edema) was measured immediately before the stimulus (zero time) and at selected time intervals following the stimulus (0.5, 1, 2, 3 and 4 h) using a plethysmometer (Panlab, Spain). The results were expressed as the variation in paw volume (mL), calculated as the difference from the basal volume.

In addition, a vascular permeability assay was carried out by intravenous (i.v.) administration of Evans’ blue (25 mg/kg). Paws were excised, weighed and incubated with 1 mL of formamide (37°C; 72 h). Plasma leakage was quantified by spectrophotometry (A_600 nm_) [16].

#### Effect of Gc-FI on rat paw edema induced by carrageenan

The paw edema induced by carrageenan was assessed according to the method of Winter et al. [17]. One hour before injection with carrageenan into the right hind paw (700 μg/paw, 100 μL, s.c, i.pl), the rats were pretreated with either sterile saline (0.9% NaCl w/v, s.c.), Gc-FI (3, 9 or 27 mg/kg, s.c.) or dexamethasone (1 mg/kg, s.c.), as a reference. The paw volume was measured immediately before (zero time) the stimulus and at selected time intervals (1, 2, 3 and 4 h) after the stimulus using a plethysmometer (Panlab, Spain). The results were expressed as the variation in paw volume (mL), calculated as the difference from the basal volume (zero time).

In addition, the extent of neutrophil accumulation in the paw tissue was measured by myeloperoxidase activity as previously described [18].

#### Evaluation of the participation of inflammatory mediators in the anti-inflammatory effect of Gc-FI

Animals were treated s.c. with Gc-FI (9 mg/kg) or sterile saline (0.9%, w/v, NaCl) 1 h before the injection of histamine (100 μg/paw, 100 μL), serotonin (20 μg/paw, 100 μl), compound 48/80 (10 μg/paw, 100 μL), bradykinin (30 μg/paw, 100 μL) or L-arginine (15 μg/paw, 100 μL). L-NAME (25 mg/kg, i.v.) was used as the standard drug for L-arginine edema. To induce the release of mast cell mediators, animals were treated intraperitoneal (i.p.) with compound 48/80 for 4 days (0.6 mg/kg on the first 3 days and 1.2 mg/kg on day 4) [19]. On the 5th day, paw edema was induced with carrageenan (700 μg/paw, intraplantar—i.pl.) in animals treated with or without compound 48/80. Edema was measured using a plethysmometer (Panlab, Spain) before (zero time) stimuli and at selected time intervals following the stimuli (0.5, 1, 2, 3 and 4 h); thereafter, edema was calculated as the difference in paw volume displacement (mL).

### Histopathological analysis

For histological examination, paw biopsies were taken 4 h after edema was induced by carrageenan. Tissue slices were fixed in 10% neutral-buffered formaldehyde, embedded in paraffin, and sectioned. The sections were stained with hematoxylin and eosin (H & E) [20]. For the specimens, histological analysis was reported as a 0–5 score grade based on the edema and inflammatory cell infiltrate observed in epithelial and connective tissues. The degree of inflammation was evaluated by two different pathologists who were blinded to the treatments. The scale was defined as follows: 0 = no inflammation, 1 = mild inflammation, 2 = mild/moderate inflammation, 3 = moderate inflammation, 4 = moderate/severe inflammation, and 5 = severe inflammation. Discrepancies in scoring were resolved by discussion, with a third examiner being consulted when consensus could not be reached [21].

### Analysis of IL-1β, TNF-α and COX-2 mRNA levels by qRT-PCR

After paw edema was induced by carrageenan, the subplantar tissue was excised from the paw for use in qRT-PCR to assess IL-1β, TNF-α and COX-2 mRNA expression. tRNA was extracted by the guanidinium isothiocyanatephenol-chloroform method according to Chomczynski and Sacchi [22]. RNA concentration was assessed by spectrophotometry using the ratio of the absorbances at 260 and 280 nm, and the integrity was verified by 1% agarose gel electrophoresis containing ethidium bromide (10 mg/mL). Complementary DNA (cDNA) was reverse transcribed from 5.0 μg of total RNA using an oligo (dT) primer and SuperScript III reverse transcriptase according to the manufacturer’s instructions (Carlsbad, CA, USA). Primers were designed by PrimerBlast [23] using GenBank (NCBI) (Table 1).

**Table 1:** Category and description of primer sequences used according to the National Center for Biotechnology Information (NCBI).

Name	Symbol	Sequence	Access Number	Amplicon Size
Glyceraldehyde-3-phosphate dehydrogenase	GAPDH	F 5’- GGGGGCTCTCTGCTCCTCCC-3’	NM_017008.3	108
		R 5’- CGGCCAAATCCGTTCACACCG-3’		
Ciclooxygenase-2	COX-2	F 5’- TCCAGTATCAGAACCGCATTGCCT-3’	NM_017232.3	149
		R 5’- AGCAAGTCCGTGTTCAAGGAGGAT-3’		
Interleukin-1 beta	IL-1β	F 5’- CCCTGCAGCTGGAGAGTGTGG-3’	NM_031512.2	153
		R 5’- TGTGCTCTGCTTGAGAGGTGCT-3’		
Tumoral necrosis factor alfa	TNF-α	F 5’- AGAACAGCAACTCCAGAACACCCT-3’	NM_012675.3	148
		R 5’- ATCTCGGATCATGCTTTCCGTGCT-3’		

Glyceraldehyde-3-phosphate dehydrogenase (GAPDH) was used as a housekeeping gene.

Contamination by genomic DNA was avoided by positioning the primers at the exon-exon junction. Quantitative Real Time PCR (qRT-PCR) for gene expression was performed using a Mastercycler realplex4 and Power SYBR Green Master Mix (Foster City, CA, USA). The samples were analyzed in a total reaction volume of 20 μL, consisting of 0.1 μg of cDNA, 10 μL of 2× SYBR Green and 300 nM of forward and reverse primers. The 2^ΔΔCt^ method [24] was used to determine the relative expression, and all of the values are expressed relative to the levels in the saline group (untreated, n = 6 per group), which were arbitrarily set to 1, as the mean ± standard error mean (SEM) [25]. The mRNA level of glyceraldehyde-3-phosphate dehydrogenase (GAPDH) was used as the endogenous control (housekeeping gene).

### Immunohistochemistry assay

After paw edema was induced by carrageenan, immunohistochemistry assays for IL-1β, TNF-α and COX-2 were performed using the streptavidin-biotin method in formalin-fixed, paraffin-embedded subplantar tissue sections (5-μm thick) from rat paws, mounted on poly-L-lysine-coated microscope slides. The sections were deparaffinized and rehydrated through xylene and a graded series of alcohols. Next, the sections were immersed in a 3% hydrogen peroxide blocking solution for 10 min. The sections were then incubated overnight (4°C) with a primary rabbit anti-IL-1β, TNF-α or COX-2 antibody diluted to 1:200 and subsequently washed with phosphate-buffered saline solution (PBS). The samples were incubated with the secondary antibody, and the incubation was performed in a chromogen solution prepared with 3,3’-diaminobenzidine (DAB). The negative control sections were assessed excluding the application of the primary antibody. The parameter of positivity for the immunohistochemical marking of the antigen in all of the specimens consisted of cells that exhibited brown staining in their cytoplasm, irrespective of the intensity of the immunomarking [26].

### Evaluation of the involvement of the HO-1 pathway

Animals were treated with ZnPP IX (3 mg/kg, s.c.) (Sigma-Aldrich, St. Louis, MO, USA), followed by the administration of Gc-FI (9 mg/kg, s.c.) 60 min later. Carrageenan (700 μg/paw, 100 μL, i.pl.) was injected after 1 h [27]. The paw volume was measured before (0 h) the stimulus and at selected time intervals (1, 2, 3 and 4 h) after the stimulus using a plethysmometer (Panlab, Spain). The results were expressed as the variation in paw volume (mL), calculated as the difference from the basal volume (0).

In addition, the bilirubin levels were measured in the blood plasma using a commercial kit (Labtest; Lagoa Santa, MG, Brazil). The samples were read at 540 nm, and the results were expressed as mg bilirubin per mL of plasma [27].

### Statistical analyses

The data are presented as the mean ± standard error of mean (SEM) of six animals per group. One- or two-way analysis of variance (ANOVA) followed by Bonferroni's test was performed. The histopathological scores were submitted to the Kruskal-Wallis and Dunn’s multiple comparisons tests. Additionally, the distribution normality of each parameter was determined using the Lilliefors [Kolmogorov–Smirnov (K–S)] test for the pooled data. A P < 0.05 was taken to indicate a statistically significant difference. All of the statistical analyses were performed using GraphPad Prism version 5.01 for Windows (GraphPad Software, 1992–2007, San Diego, CA; www.graphpad.com).

## Results

### Gc-FI and Gc-FII reveal characteristics of agarocolloids

**Fig 1:**
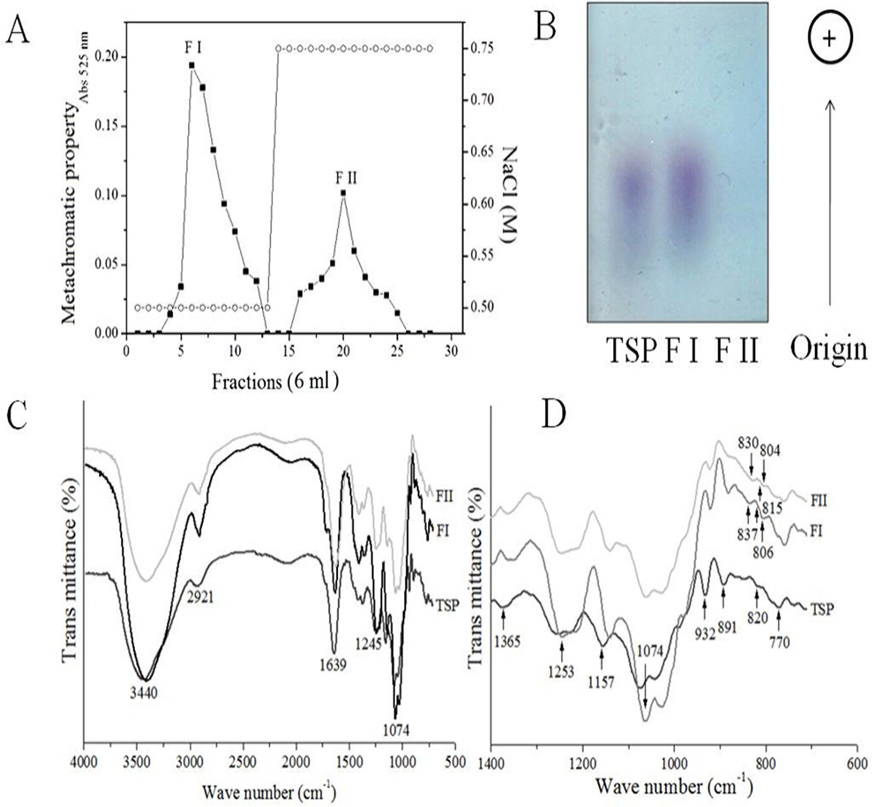
Separation of Gc-TSP by DEAE-cellulose (Fractions Gc-FI and Gc-FII). Metachromasia (■—■) and NaCl concentrations (○—○) (A). Agarose gel electrophoresis (B) and FT-IR spectra at 500 and 4000 cm^-1^ (C and D).

Anion-exchange chromatography on a DEAE-cellulose column separated the Gc-TSP into two fractions (Gc-FI and Gc-FII) when eluted with 0.5 and 0.75 M NaCl, respectively (Fig. 1A). The agarose gel electrophoresis technique revealed marked differences in the charge density between the obtained fractions. The absence of the Gc-FII fraction is justified due to low sulfate content in the analyzed polymer. Additionally, Gc-FI presented similar mobility on a gel compared with Gc-TSP, although it was polydispersed (Fig. 1B).

The FT-IR spectra of Gc-TSP, Gc-FI and Gc-FII showed typical absorption bands related to the presence of agarocolloids (signals at 1365, 1253, 1157, 1074, 922–932, 891 and 770 cm^-1^) in the analyzed polysaccharides (Figs. 1C, D). When the FT-IR spectra were extended, a decrease in the signal intensity of the ester sulfate groups between the fractions (from Gc-FI (high value) to Gc-FII) was observed. The band absorbance from the fractions revealed the occurrence of 2-sulfate galactose at 830–837 cm^-1^ and sulfate on C-2 of 3,6-anhydrogalactose at 804–806 cm^-1^. Additionally, all of these spectra displayed signals from 815 to 820 cm^-1^ related to the presence of galactose-6-sulfate (Fig. 1D).

### Gc-FI presents anti-inflammatory effects by different mediators

**Fig 2:**
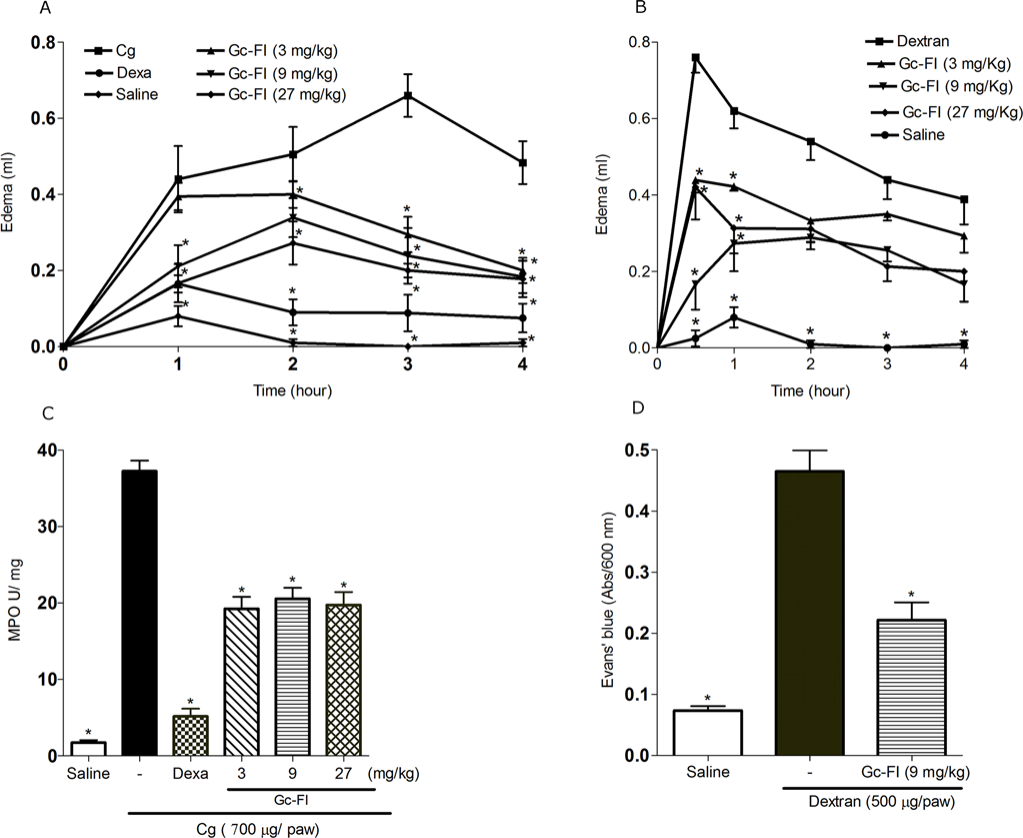
Effect of Gc-FI on paw edema induced by Cg (A) or dextran (B). MPO activity (C) and vascular permeability (D). Mean ± SEM (%, n = 6) (ANOVA, Bonferroni’s test). * p < 0.05 or p < 0.001 compared with the stimulus.

Carrageenan induced intense paw edema, which reached a maximum level at 3 h (0.66 ± 0.05 mL) after injection. Gc-FI (3, 9 or 27 mg/kg, s.c.) significantly reduced edema formation, particularly in the third hour, with a reduction of 56.0% (0.29 ± 0.04 mL), 63.6% (0.24 ± 0.07 mL) and 65.0% (0.23 ± 0.03 mL), respectively. Dexamethasone (1 mg/kg, s.c.) also inhibited edema by 86.5% (0.08 00B1 0.04 mL) (Fig. 2A). The data were confirmed by MPO activity, which indicated that Gc-FI (3, 9 or 27 mg/kg, s.c.) (p < 0.001) inhibited neutrophil accumulation in the paw by 48.5, 44.7 and 47%, respectively. Dexamethasone also inhibited MPO activity (86%) (Fig. 2C). Dextran induced intense paw edema and had a maximum effect at 0.5 h (0.76 ± 0.04 mL) after the administration that decreased over the subsequent hours. The pretreatment (s.c.) of animals with different doses of Gc-FI (3, 9 or 27 mg/kg) inhibited (p < 0.05) paw edema induced by dextran at 0.5 h by 56.1% (0.43 ± 0.03 mL), 70.5% (0.18 ± 0.07 mL) and 44.7% (0.42 ± 0.08 mL), respectively (Fig. 2B). Additionally, Gc-FI (9 mg/kg, s.c.) reduced (p < 0.001) the vascular permeability induced by dextran (0.43 ± 0.03 mL) by 48.9% (0.22 ± 0.02 mL) (Evan’s blue assay) (Fig. 2D).

**Fig 3:**
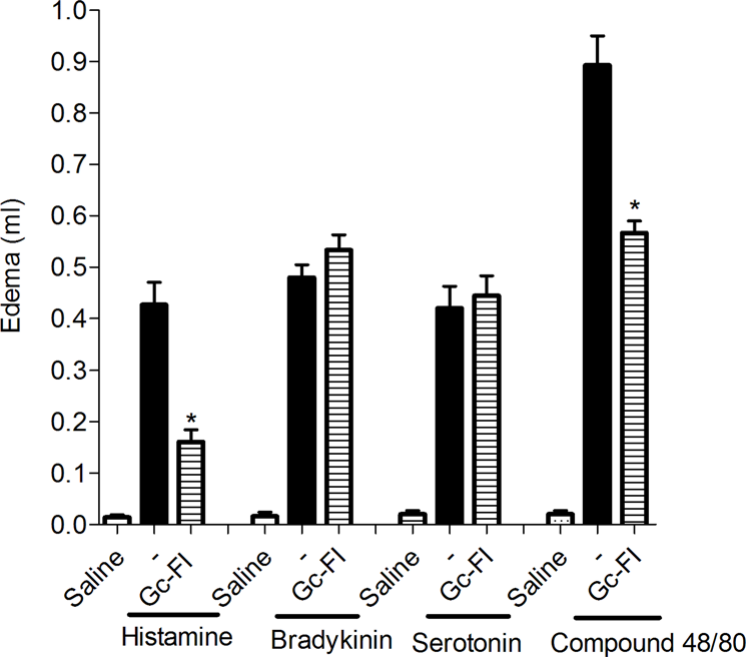
The anti-inflammatory effect of Gc-FI involves the inhibition of histamine and compound 48/80. Mean ± SEM (%, n = 6) (ANOVA, Bonferroni’s test). * p < 0.05 compared to stimuli.

Gc-FI (9 mg/kg, s.c.) significantly reduced the edematogenic effect elicited by histamine (0.42 ± 0.04 mL) and compound 48/80 (0.90 ± 0.05 mL), at 0.5-h intervals, by approximately 63% (0.16 ± 0.02 mL) and 45% (0.59 ± 0.03 mL), respectively, but had no effect on bradykinin and serotonin-induced paw edemas (Fig. 3).

**Fig 4:**
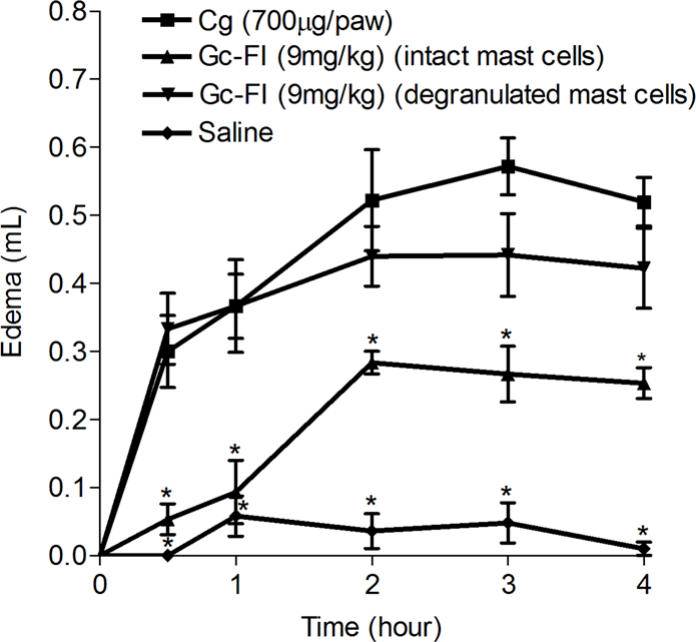
Effect of Gc-FI on Cg-induced paw edema in rats with intact mast cells. Mean ± S.E.M. (%, n = 6) (ANOVA, Bonferroni tests).* p < 0.05 compared with the stimulus.

Moreover, Gc-FI significantly inhibited carrageenan-induced edema in animals with intact mast cells, at all intervals, compared with the carrageenan group but was ineffective in reducing edema in animals with degranulated mast cells (Fig. 4).

Gc-FI also inhibited (p < 0.001) paw edema produced by L-arginine (0.42 ± 0.07 mL), a substrate of NO generation, by 76.2% (0.10 ± 0.01 mL). This property was also observed for the standard inhibitor L-NAME (30 mg/kg, i.p.) (71.5%, 0.12 ± 0.04 mL) (Table 2).

**Table 2:** Effect of Gc-FI on L-arginine-induced paw edema in rats.

Experimental groups	Paw edema (mL)									
	0.5h	1h	2h	3h	4h
Saline	0.01 ± 0.01*	0.05 ± 0.01*	0.01 ± 0.01*	0.00 ± 0.00*	0.01 ± 0.01*
L-arginine	0.12 ± 0.04	0.22 ± 0.03	0.36 ± 0.05	0.38 ± 0.06	0.30 ± 0.05
Gc-FI	0.08 ± 0.03	0.06 ± 0.03*	0.08 ± 0.05*	0.10 ± 0.01*	0.09 ± 0.04*
L-NAME	0.11 ± 0.02	0.08 ± 0.02*	0.13 ± 0.03*	0.12 ± 0.04*	0.07 ± 0.02*

Mean ± S.E.M. (%, n = 6). (ANOVA, Bonferroni tests).

* p < 0.001 compared to stimulus.

### Gc-FI reverses tissue alterations in the carrageenan-inflamed paw as assessed by H & E staining

The degree of inflammation was evaluated by inflammation scores from 0 to 5, which were determined based on the parameters for cellular infiltration and edema (Table 3).

**Table 3:** Histopathological analysis (scores) of subplantar tissues after treatment s.c. rats with Gc-FI.

Groups	Inflammatory cells	Edema
Saline	0 (0–0)*	0 (0–0)*
Carrageenan	5 (4–5)	4 (3–5)
Gc-FI (3 mg/kg)	2 (1–3)*	2 (2–4)*
Gc-FI (9 mg/kg)	1 (0–1)*	0 (0–1)*
Gc-FI (27 mg/kg)	1 (0–2)*	1 (0–1)*

**Fig 5:**
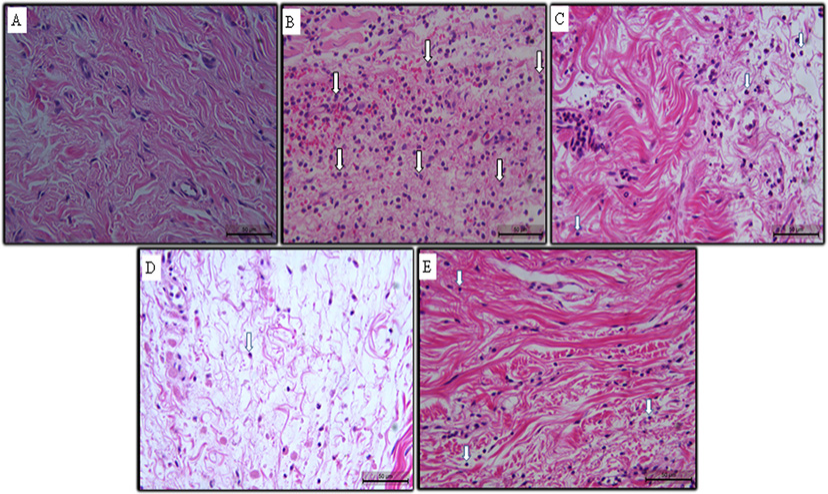
Photomicrographs of the histopathological analysis of paw tissue from rats treated with Gc-FI (3, 9 or 27 mg/kg): (a) saline (b) Cg (c) 3 mg/kg (d) 9 mg/kg (e) 27 mg/kg. White arrows: neutrophils. Scale bar: 50 μm.

The saline control group showed normal subplantar tissue histology (Fig. 5A). By contrast, carrageenan injection into the rat right hind paw caused massive accumulation of infiltrated inflammatory cells and edema formation (Fig. 5B). The cell types present in the carrageenan group after the induction of inflammation were predominantly polymorphonuclear leukocytes (neutrophils), reflecting acute inflammation. However, the infiltration of inflammatory cells and edema were significantly decreased following treatment with Gc-FI (Figs. 5C, D and E), confirming the data observed in dextran- and carrageenan-induced paw edema.

### Gc-FI down-regulates the carrageenan-induced IL-1β, TNF-α and COX-2 mRNA levels on subplantar tissues from the rat paw

**Fig 6:**
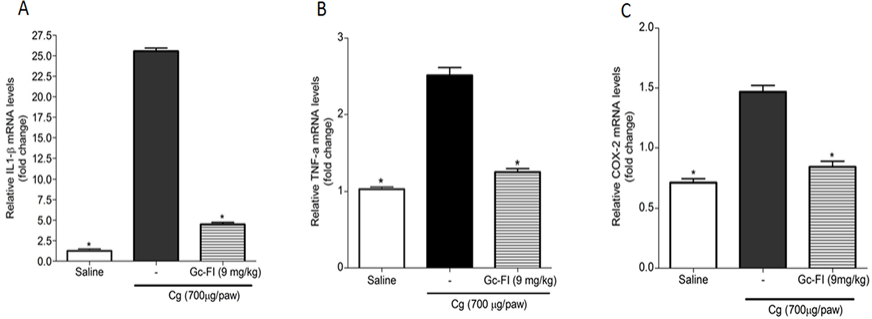
Effect of Gc-FI (9 mg/kg, s.c.) on the expression levels of IL-1β (A), TNF-α (B) and COX-2 (C). * p < 0.05 compared with the Cg group (ANOVA, Bonferroni test).

qRT-PCR analysis revealed (p < 0.05) down-modulation of the TNF-α (1.252 ± 0.047-fold), COX-2 (0.939 ± 0.016-fold) and IL-1β (4.478 ± 0.253-fold) mRNA levels in the group treated with Gc-FI (9 mg/kg, s.c.) compared with the carrageenan group (TNF-α: 2.245 ± 0.215-fold; COX-2: 1.412 ± 0.054-fold; IL-1 β: 25.563 ± 0.368-fold), respectively (Fig. 6A, B and C). This down-regulation was confirmed by protein expression in the immunohistochemistry assay.

### Gc-FI decreases IL-1β, TNF-α and COX-2 immunoexpression in carrageenan-induced paw edema in rats

**Fig 7:**
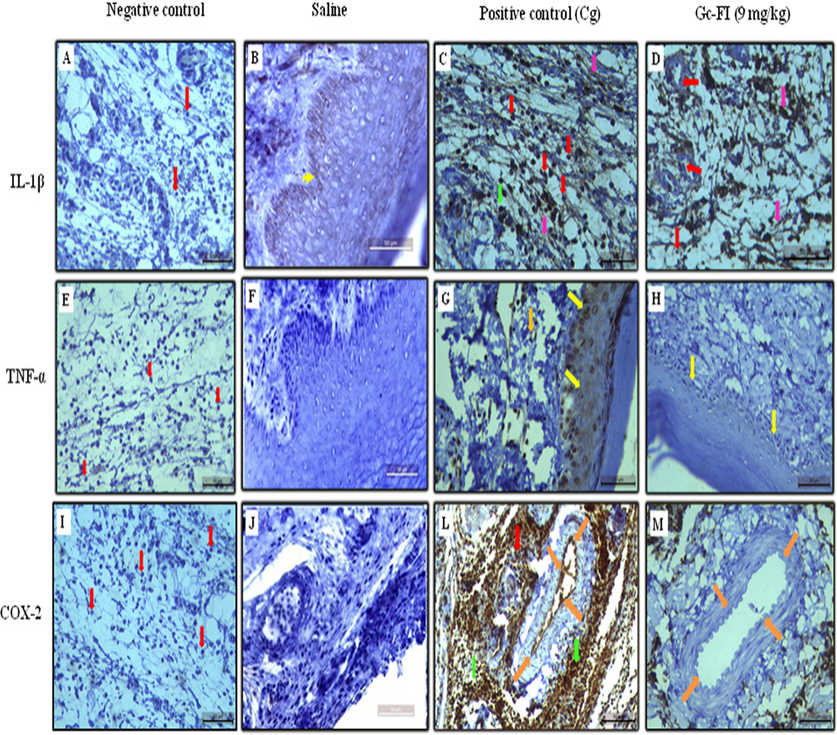
Immunohistochemical staining for IL-1β, TNF-α and COX-2 expression in paw tissues. Negative control (A, E, I), saline (B, F, J), positive control (C, G, L) and 9 mg/kg Gc-FI (D, H, M). Red arrows: neutrophils. Yellow arrows: epithelium. Green arrows: lymphocyte. Purple arrows: extracellular matrix. Orange arrows: endothelium cell. Scale bar: 50 μm.

Immunohistochemistry analysis showed intense immunostaining of IL-1β in the extracellular matrix and some cells in the paw conjunctive tissue, mainly neutrophils and lymphocytes, in rats subjected to carrageenan-induced paw edema (Fig. 7C) compared with that in the saline control (Fig. 7B). This increased immunostaining of IL-1β was characterized by brown staining in the cells and extracellular matrix. In the carrageenan group, immunostaining for IL-1β was markedly reduced after treatment with Gc-FI (9 mg/kg, s.c.). Additionally, leukocyte infiltration was reduced (measured by neutrophils into the vessel), and a small number of neutrophils present in conjunctive tissue showed no immunostaining, indicating that Gc-FI reduced the expression of IL-1β in this acute inflammation model in rats (Fig. 7D). No staining was observed in the sample that did not receive the primary antibody (negative control) (Fig. 7A).

The negative control group showed no immunostaining for TNF-α (Fig. 7E). However, the carrageenan group showed intense immunostaining of TNF-α in the epithelium and strong brown staining in the endothelial cells (Fig. 7G) compared with that in the saline control (Fig. 7F). Regarding Gc-FI (9 mg/kg, s.c.), the number of cells immunostained for TNF-α was significantly decreased in both tissues (Fig. 7H). The negative and saline control groups showed no immunostaining for COX-2 (Figs. 7I and J). However, the carrageenan group showed intense immunostaining of COX-2 in some cells in the paw conjunctive tissue, mainly neutrophils, lymphocytes and endothelial cells (Fig. 7L), compared with the animals receiving saline (Fig. 7J). Interestingly, pre-treatment with Gc-FI (9 mg/kg, s.c.) drastically reduced the immunostaining for COX-2 because no immunostaining of endothelial cells was observed (Fig. 7M) compared with that in the carrageenan group (Fig. 7L).

### Gc-FI also presents anti-inflammatory action by the HO-1 pathway

**Fig 8:**
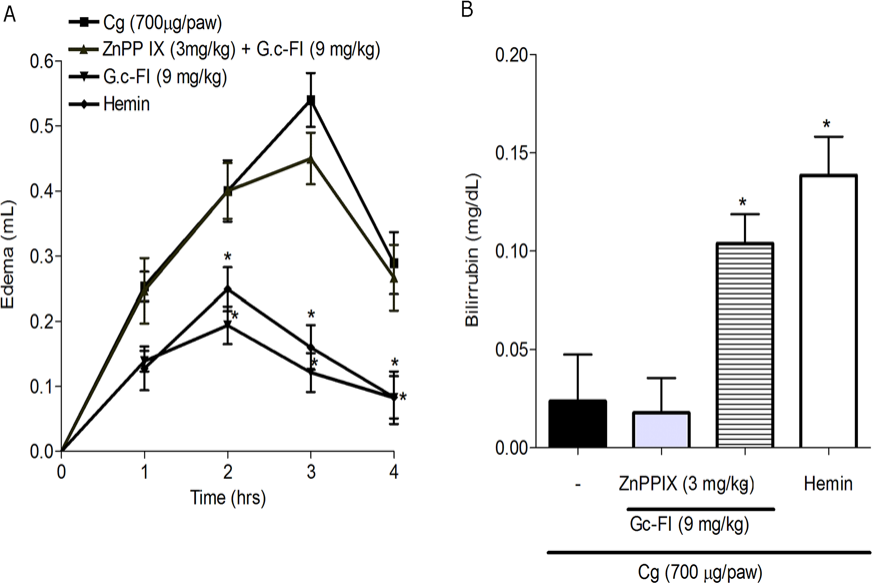
HO-1 activity in paw edema induced by Cg (A) and bilirubin levels (mg/dl) in the blood plasma of rats after pretreatment with ZnPP IX (B). Data are expressed as the mean ± S.E.M of six rats for each group. * p < 0.05 from the Cg group (ANOVA, Bonferroni’s test).

Gc-FI (9 mg/kg, s.c.), after s.c. pretreatment with ZnPPI, did not inhibit the edema induced by carrageenan compared with that in the group that received only Gc-FI as pretreatment. Moreover, pretreatment with only hemin (a HO-1 substrate) also inhibited the edema induced by carrageenan (Fig. 8A).

The bilirubin levels in the blood plasma were reduced by pretreatment with ZnPP IX, while pretreatment with only Gc-FI (9 mg/kg, s.c.) significantly increased the bilirubin level compared with pretreatment with ZnPP IX. Similarly, pretreatment with hemin only also significantly increased the bilirubin levels (Fig. 8B).

## Discussion

In the present study, the chromatographic profile of Gc-TSP was similar to that of TSP from *Gracilaria birdiae*, which also contained two polysaccharidic fractions. However, the physicochemical characteristics of Gc-FI and Gc-FII observed in the present investigation were distinct from those previously reported for *G*. *birdie* [28]. Gc-FI was also a polydisperse band as detected by agarose gel electrophoresis (Fig. 1B), an observation that is typical of sulfated polysaccharides from seaweeds [28], [29], [30]. Additionally, in the IR spectra, both Gc-FI and Gc-FII, which exhibited a difference in the intensity of signals related to the presence of ester sulfate groups, contained agar, confirming the agar-type polysaccharide (Fig. 1C) [11]. Interestingly, the detected absorption bands at the positions of 2-sulfate galactose and C-2 of 3,6-anhydrogalactose in the spectra from these fractions (Fig. 1D) revealed a new insight in the study of *G*. *cornea*. These data explained why these fractions exhibited important differences in their relative proportions of sulfate after the preliminary physicochemical characterization by electrophoresis [31].

Carrageenan-induced rat paw edema is a classic test for examining the effectiveness of anti-inflammatory agents [17]. This phlogistic agent evokes biphasic edema, the first phase of which is mediated by the release of histamine and serotonin from mast cells and the second phase of which involves neutrophil infiltration and the release of prostaglandin E2, cytokines (mainly IL-1β) and NO [1], [32]. Our results demonstrated that Gc-FI significantly reduced both phases of the carrageenan-induced edema (Fig. 2A) and neutrophil migration, as confirmed by the MPO activity in the subplantar tissue from the rat paw (Fig. 2C) that is commonly considered a biochemical indicator of neutrophil infiltration [33]. The data were also supported by the histopathological analysis (Fig. 5). Previous studies have demonstrated that sulfated polysaccharidic fractions obtained from other *Gracilaria* species showed an anti-inflammatory effect in carrageenan-induced paw edema [28], [34].

The dextran-induced paw edema model is characterized by increased vascular permeability, the activation of kinins, and the release of histamine and serotonin from mast cells, leading to osmotic edema with low levels of protein and neutrophils [15]. Gc-FI could inhibit, in all of the doses tested, the dextran-induced paw edema and reduce the increase in vascular permeability induced by dextran (Figs. 2B, D). These observations were supported by the literature, in which a sulfated polysaccharidic fraction from the red seaweed *Gelidium crinale* inhibited not only the dextran-induced paw edema but also the increase in vascular permeability, when verified by the Evans’ blue assay [35].

Gc-FI inhibited the paw edema induced by both dextran and carrageenan (early and late phases). It could be postulated that the Gc-FI anti-inflammatory effect is associated with the inhibition of cellular and vascular events, reinforcing the *in vivo* effects found for Gc-TSP from the same algal species in another study [10].

To clarify this mechanism, different inflammatory inductors were used in the paw edema model, using Gc-FI at the dose of 9 mg/kg s.c. to present the best anti-inflammatory result. Gc-FI (9 mg/kg, s.c) inhibited the paw edema induced by histamine but did not reduce the edema induced by serotonin and bradykinin (Fig. 3). Similarly, de Sousa et al. [35] investigated a sulfated polysaccharidic fraction from the red seaweed *G*. *crinale* and reported an anti-edematogenic response by the inhibition of histamine-induced rat paw edema but not by inhibition of the serotonin-induced rat paw edema.

Because our results showed that Gc-FI (9 mg/kg, s.c.) inhibited the paw edema induced by histamine, the effect of Gc-FI on the histamine released from the mast cells was further tested. To clarify this suggestion, compound 48/80-induced paw edema was performed. It has been described that compound 48/80 induces the release of histamine and serotonin from mast cells, eliciting an edematogenic effect in rats [4], [19].

In the present study, Gc-FI (9 mg/kg, s.c.) inhibited the paw edema induced by compound 48/80 (Fig. 3). De Sousa et al. [35] reported that a sulfated polysaccharidic fraction from the red seaweed *G*. *crinale* could reduce compound 48/80-induced rat paw edema.

Based on this finding, Gc-FI (9 mg/kg, s.c.) could be stabilizing mast cell membranes, preventing its content release [4]. To confirm this hypothesis, compound 48/80-induced *in vivo* mast cell degranulation was performed. In fact, Gc-FI reduced both phases of carrageenan-induced paw edema in animals with intact mast cells but was ineffective in reducing edema compared with compound 48/80 treatment (degranulated mast cells) (Fig. 4). This study is the first report describing the protective role of mast cell membranes in the anti-inflammatory process modulated by sulfated polysaccharides from seaweeds. It is possible to postulate that histamine is the major target of the Gc-FI anti-inflammatory effect, reinforcing its role in the osmotic edema induced by dextran and in the initial phase of that induced by carrageenan [29].

The participation of late-phase mediators was also suggested for the Gc-FI anti-inflammatory effect. Gc-FI (9 mg/kg, s.c.) reduced the paw edema induced by L-arginine in a manner similar to L-NAME (Table 2), a non-selective inhibitor of NO synthase. A similar role was also observed for a sulfated polysaccharidic fraction from the brown seaweed *Lobophora variegata* by Siqueira et al. [30].

The late edema phase induced by carrageenan is known to be dependent on cytokine production by resident cells and neutrophils [36]. IL-1β and TNF-α are potent pro-inflammatory cytokines that have multiple effects, including the activation of inflammatory cells, induction of several inflammatory proteins, cytotoxicity, edema formation, and neutrophil migration [37], [38]. From these reports, it could be inferred that the anti-inflammatory effect of Gc-FI might occur through the inhibition of the cytokines involved in carrageenan-induced paw edema, as described for the anti-inflammatory mechanism of indomethacin in inhibiting the inflammatory process, induced by carrageenan [36]. In fact, Gc-FI caused a relative decrease in the IL-1β and TNF-α mRNA levels in the groups that were previously treated with Gc-FI (9 mg/kg, s.c.) comparison with those in the carrageenan group (Fig. 6A, B), as confirmed by immunohistochemistry (Fig. 7). Chaves et al. [34] demonstrated that a sulfated polysaccharidic fraction from the red seaweed *Gracilaria caudata* reduced IL-1β and TNF-α levels in a peritonitis model induced by carrageenan in rats.

Gc-FI was also capable of reducing the COX-2 mRNA levels in subplantar tissue from the rat paw compared with those in the carrageenan group (Fig. 6C). Similarly, this trend was observed for the protein expression of COX-2 (Fig. 7H) and indicated that Gc-FI inhibits the production of PGE2, a degradation metabolite of arachidonic acid, via the inhibition of COX-2 expression. This finding reinforces the anti-edematogenic effect of Gc-FI in the late phase of carrageenan-induced paw edema (Fig. 2A) because, in this phase, edema is also sustained by PGE2 released via COX-2 [36], [39].

Several lines of evidence have suggested an involvement of HO-1 in the anti-inflammatory activity of cells and tissues [40–41]. Studies have demonstrated that HO-1 expression and the concomitant production of its metabolites (CO and biliverdin/bilirubin) can reduce edema, leukocyte adhesion, migration and the production of cytokines [42]. After pretreatment with ZnPP IX, an anti-inflammatory effect of Gc-FI on carrageenan-induced paw edema was not observed (Fig. 8A). This result indicated that the HO-1 pathway is associated with the anti-inflammatory mechanism of Gc-FI in this model. According to Vanderlei et al. [28], the anti-inflammatory effect of a sulfated polysaccharidic fraction from the red seaweed *G*. *birdiae* is associated with the integrity of the HO-1/biliverdin/bilirubin/CO pathway. Accordingly, Ribeiro et al. [43] reported that, after pretreatment with ZnPP IX, no anti-inflammatory effect of a sulfated polysaccharidic fraction from the green seaweed *Caulerpa racemosa* on carrageenan-induced paw edema was observed.

Subsequently, it was observed that the bilirubin levels were reduced by pretreatment with ZnPP IX, while pretreatment with only Gc-FI significantly increased the bilirubin levels; therefore, HO-1 activity was enhanced. Grangeiro et al. [44] determined the bilirubin levels in peritoneal exudates, as indexes of the HO-1 activity, in the writhing test. After an acetic acid challenge, increased levels of bilirubin were detected in the peritoneal exudate, and ZnPP IX reduced bilirubin production. Hence, these findings further reinforced the participation of this pathway in the anti-inflammatory effects of Gc-FI.

Our results show that the anti-inflammatory mechanism of Gc-FI is very complex. In fact, the cellular and molecular mechanism of the anti-inflammatory effect might be a combination of targeting multiple points involved in the inflammatory phenomenon. The lack of structure-function relationship data makes the understanding of the anti-inflammatory role of sulfated polysaccharides difficult [45].

**Fig 9:**
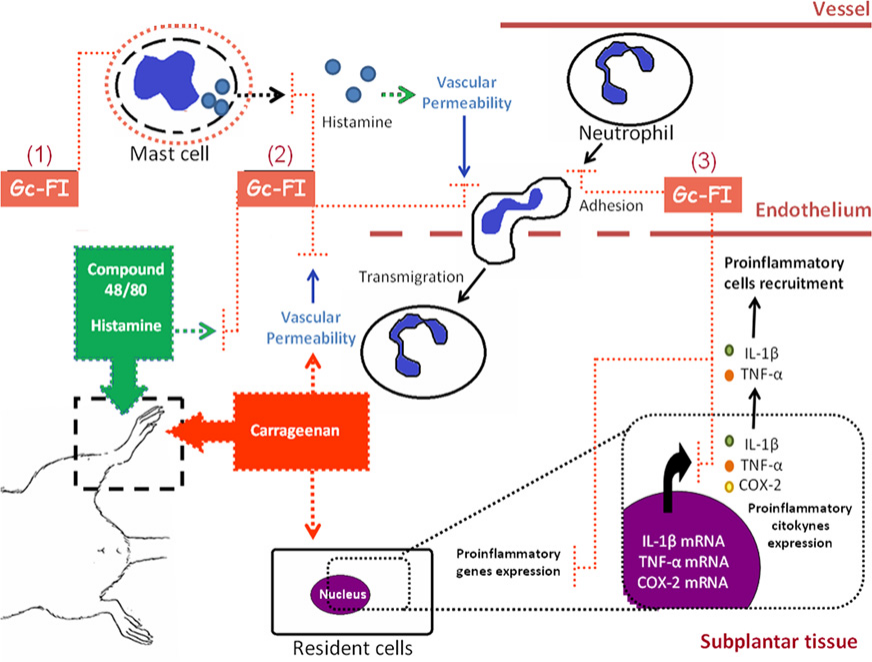
The anti-inflammatory partial mechanism of Gc-FI.

We proposed a partial anti-inflammatory mechanism of Gc-FI in which it would play a regulatory role via three ways: (1) stabilization of the mast cell membrane; (2) antihistaminic action; and (3) down-regulation in the production of proinflammatory mediators (Fig. 9). The HO-1/bilirubin pathway would also be involved in the Gc-FI anti-inflammation response; however, complementary studies based on these involved inflammatory mediators should be explored.

## Conclusions

A sulfated polysaccharidic fraction from the red seaweed *Gracilaria cornea* presents anti-inflammatory action via modulation of the acute inflammatory process by inhibition of histamine, vascular permeability and neutrophil migration. Its action is also related to IL-1β, TNF-α and COX-2 down-modulation. This study showed a relevant protective role of these molecules on the mast cell membrane and possible involvement of the HO-1 pathway.
